# Proteomic analysis across patient iPSC-based models and human post-mortem hippocampal tissue reveals early cellular dysfunction and progression of Alzheimer’s disease pathogenesis

**DOI:** 10.1186/s40478-023-01649-z

**Published:** 2023-09-15

**Authors:** Yuriy Pomeshchik, Erika Velasquez, Jeovanis Gil, Oxana Klementieva, Ritha Gidlöf, Marie Sydoff, Silvia Bagnoli, Benedetta Nacmias, Sandro Sorbi, Gunilla Westergren-Thorsson, Gunnar K. Gouras, Melinda Rezeli, Laurent Roybon

**Affiliations:** 1 iPSC Laboratory for CNS Disease Modelling, Department of Experimental Medical Science, BMC D10, Lund University, 22184 Lund, Sweden; 2 Strategic Research Area MultiPark, Lund University, 22184 Lund, Sweden; 3 Lund Stem Cell Center, Lund University, 22184 Lund, Sweden; 4 Clinical Protein Science & Imaging, Department of Biomedical Engineering, BMC D13, Lund University, 22184 Lund, Sweden; 5 Medical Micro-Spectroscopy, Department of Experimental Medical Science, BMC B10, Lund University, 22184 Lund, Sweden; 6 Lund University BioImaging Centre, Faculty of Medicine, Lund University, 22142 Lund, Sweden; 7 Laboratorio Di Neurogenetica, Dipartimento Di Neuroscienze, Psicologia, Area del Farmaco e Salute del Bambino- NEUROFARBA, Università degli Studi di Firenze, 50134 Florence, Italy; 8 IRCCS Fondazione Don Carlo Gnocchi, Florence, Italy; 9 Department of Experimental Medical Science, BMC C12, Faculty of Medicine, Lund University, 22142 Lund, Sweden; 10 Experimental Dementia Research Unit, Department of Experimental Medical Science, BMC B11, Lund University, 22184 Lund, Sweden; 11 Swedish National Infrastructure for Biological Mass Spectrometry (BioMS), Lund University, 22184 Lund, Sweden; 12 Department of Neurodegenerative Science, The MiND Program, Van Andel Institute, Grand Rapids, MI USA

**Keywords:** Induced pluripotent stem cells, Alzheimer’s disease, Hippocampal spheroids, Intracerebral transplantation, Proteomic analysis, Human post-mortem tissue

## Abstract

The hippocampus is a primary region affected in Alzheimer’s disease (AD). Because AD postmortem brain tissue is not available prior to symptomatic stage, we lack understanding of early cellular pathogenic mechanisms. To address this issue, we examined the cellular origin and progression of AD pathogenesis by comparing patient-based model systems including iPSC-derived brain cells transplanted into the mouse brain hippocampus. Proteomic analysis of the graft enabled the identification of pathways and network dysfunction in AD patient brain cells, associated with increased levels of Aβ-42 and β-sheet structures. Interestingly, the host cells surrounding the AD graft also presented alterations in cellular biological pathways. Furthermore, proteomic analysis across human iPSC-based models and human post-mortem hippocampal tissue projected coherent longitudinal cellular changes indicative of early to end stage AD cellular pathogenesis. Our data showcase patient-based models to study the cell autonomous origin and progression of AD pathogenesis.

**Graphical Abstract:**

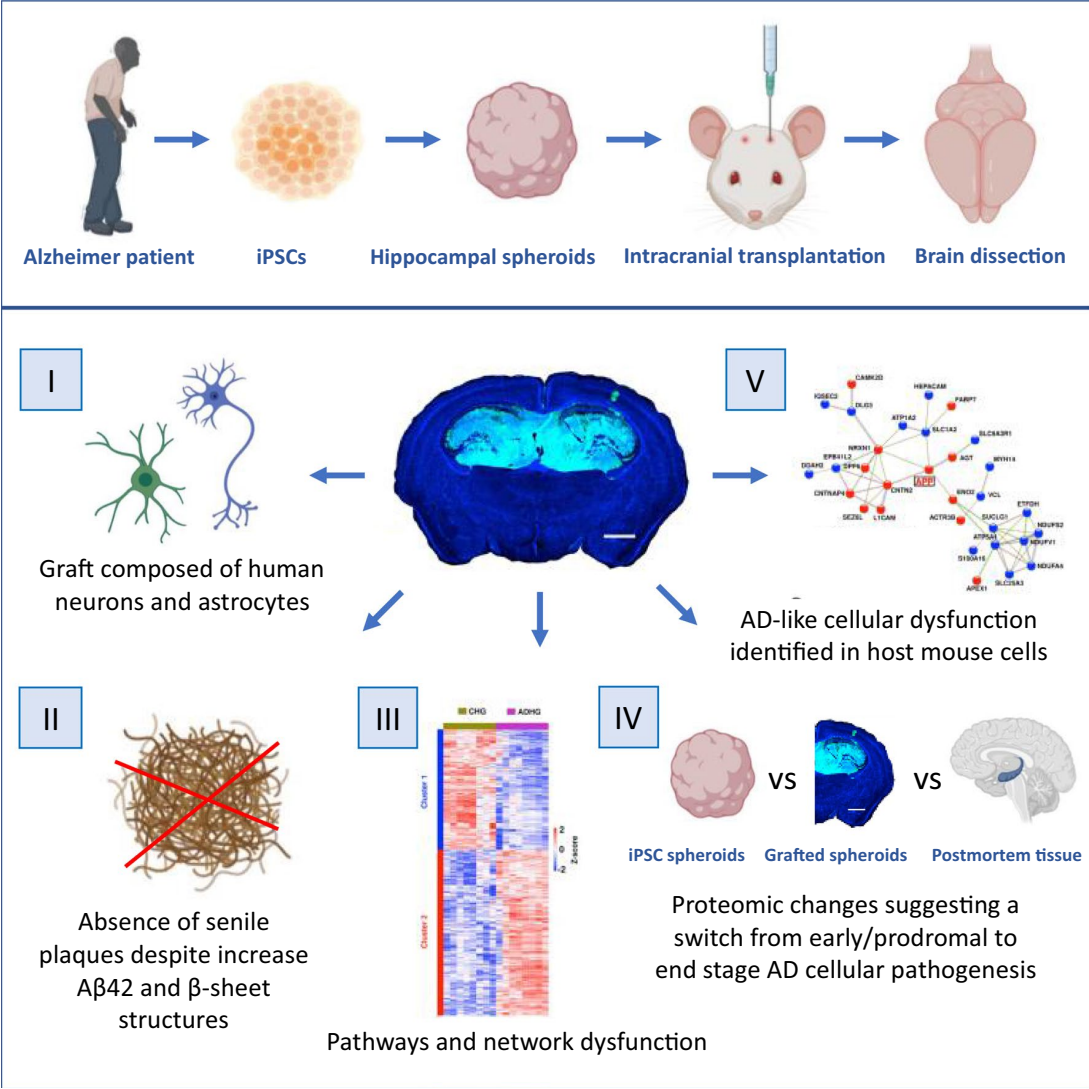

**Supplementary Information:**

The online version contains supplementary material available at 10.1186/s40478-023-01649-z.

## Background

Alzheimer’s disease (AD) is a progressive neurodegenerative disorder of the brain and the most common form of dementia. Pathological hallmarks are amyloid plaques mainly composed of amyloid-β (Aβ) 42 peptides and neurofibrillary tangles consisting of abnormally phosphorylated tau proteins in AD brain tissue [1]. While familial forms of AD have been identified, most cases have an unknown aetiology.

Transgenic animal models overexpressing gene variants associated with AD have uncovered putative mechanisms of cellular pathology [2]. However, these models cannot fully inform on mechanisms underlying early cellular dysfunctions and their progression in patient brain cells, which is needed to successfully develop therapies. Reprogramming of patient-derived somatic cells into induced pluripotent stem cells (iPSCs) [3, 4] has emerged as a powerful tool to model familial and idiopathic AD cellular pathogenesis [5–10]. The cells are human, they carry the genetic makeup of the individual they are generated from, and they are young (embryonic-like). This suggests that cellular phenotypes identified in patient iPSC-derived brain cells could inform about early/prodromal stages of AD.

Numerous studies have demonstrated that iPSC-derived brain cells, in particular cortical neurons, grown in vitro display molecular and cellular changes associated with AD pathology [11–13]. We showed that iPSC-derived hippocampal neurons carrying the amyloid precursor protein (APP) p.V717I pathogenic variant, exhibited significant alterations in cellular pathways and networks, coupled to increased intracellular and extracellular Aβ42/40 peptide ratios, synaptic dysfunction, and β-sheet structure formation [7]. A limitation of these studies was that the iPSC-derived brain cells were maintained in an artificial cultured environment, which does not closely mimic the brain environment, despite the cells sometimes being grown as spheroids. This important issue was initially addressed by the Goldman group and circumvented by the generation of humanized models, to experimentally study cell autonomous phenotypes within schizophrenia and Huntington’s disease patient glia, in the brain of living mice [14, 15]. A human-rodent chimeric model was recently developed to experimentally study cellular toxicity induced by ApoE4, the strongest genetic risk factor associated with late onset AD, in human cortical neurons in vivo [16]. Additionally, two studies investigated how AD pathology would affect human pluripotent stem cell-derived neurons and astrocytes following transplantation in the AD mouse brain [17, 18]. Lacking however, is an understanding of cell autonomous dysfunctions in AD patient iPSC-derived brain cells grown in vivo, and how they differ from those present in AD patient iPSC-derived brain cells grown in vitro for 100 days and in brain cells at end stage of AD.

Here, we generated a chimeric model of AD by transplanting AD patient iPSC-derived hippocampal brain cells into the hippocampi of immunodeficient mice. We analyzed cellular pathogenesis in the grafted cells and host tissue 6 months post-engraftment, using advanced imaging, biochemical and liquid chromatography-tandem mass spectrometry (LC–MS/MS) techniques. Finally, we compared the cellular pathways and network dysfunction of the grafted AD cells with those present in AD patient iPSC-derived hippocampal neurons grown in vitro and AD post-mortem hippocampal tissue.

## Results

### Human iPSC-derived hippocampal brain cells survive in the mouse brain and express neuronal and astrocytic markers

**Fig. 1:**
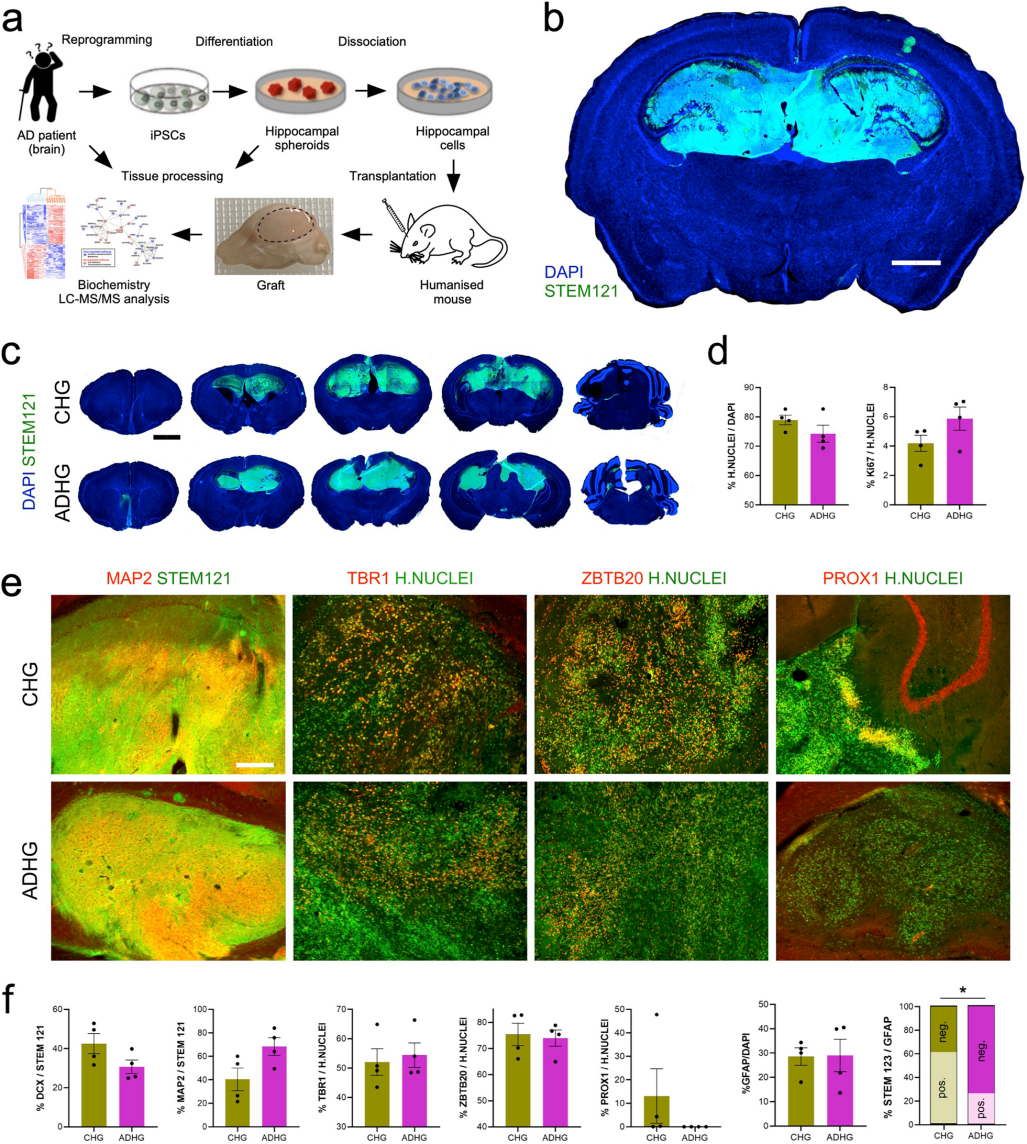
Characterization of the iPSC-derived human grafts (HG) in the mouse brain **a** Schematic representation of the experimental workflow. **b** and **c** Immunostaining for human cytoplasmic marker STEM121 and nuclear marker DAPI in control and AD grafts six months after transplantation into mouse hippocampus. Scale bars: 1 mm (b) and 2 mm (c). ADHG: AD human graft; CHG: Control human graft. **d** Quantification of human Nuclei marker-positive cells expressed relative to the total number of DAPI-labeled cells and Ki-67-positive cells expressed relative to the total number of human Nuclei marker-positive cells in control and AD iPSC-derived human grafts. Results are presented as mean ± S.E.M. N = 4 animals. Statistical analysis by two-tailed t-test. **e** Immunostaining for human Nuclei marker and neuronal markers MAP2, TBR1, and hippocampal markers ZBTB20 and PROX1 in control and AD iPSC-derived human grafts. Scale bars: 200 µm. **f** Quantification of DCX-, MAP2-, TBR1-, ZBTB20-, PROX1-, GFAP- and STEM123-positive cells in control and AD iPSC-derived human grafts. Results are presented as mean ± S.E.M. N = 4 animals. P value: ∗ p < 0.05. Statistical analysis by two-tailed t-test

To investigate the effect of an APP pathogenic variant in human hippocampal brain cells in vivo, we differentiated iPSCs lines generated from a non-demented female individual and an AD female patient carrying the most common missense variation of the APP gene (APP p.V717I), into hippocampal spheroids [7]. Fifty-day old spheroids containing hippocampal neurons and neural progenitors were dissociated into single cells and transplanted into the hippocampi of 3-month-old Rag2 immunodeficient mice. Immunohistochemical analysis of the graft 6-month post-transplantation revealed that the human cells had survived and integrated in the host hippocampi (Fig. 1b–d). We estimated that 5% of all human cells were still actively dividing (Fig. 1d and Additional file 1: Figure S1). Amongst the human grafted cells, 40–65% and 30–40% stained positive for MAP2 and Doublecortin, respectively (Fig. 1e, f). While most human neurons expressed T-Box Brain Transcription Factor 1 (TBR1) and Zinc Finger and BTB Domain Containing 20 (ZBTB20) markers of hippocampal identity, few were positive for PROX1 (Fig. 1e, f). Immunohistochemistry for STEM123 and ionized calcium binding adaptor molecule 1 (IBA1) revealed the presence of human astrocytes and the absence of human microglia, respectively (Fig. 1f and Additional file 1: Figure S1). Interestingly, the number of host astrocytes was greater in the AD hippocampal grafts (ADHG), compared to control hippocampal grafts (CHG). It is worth mentioning that as opposed to mouse astrocytes, the number of mouse microglia inside the graft did not differ between AD and control grafts (Additional file 1: Figure S1).

Together, these data show that the grafts were composed of human neuroblasts, neurons and astrocytes, and that neurogenesis was still ongoing 6-month post-transplantation.

### AD cellular pathology identified in 6-month-old graft despite absence of senile plaques

**Fig. 2:**
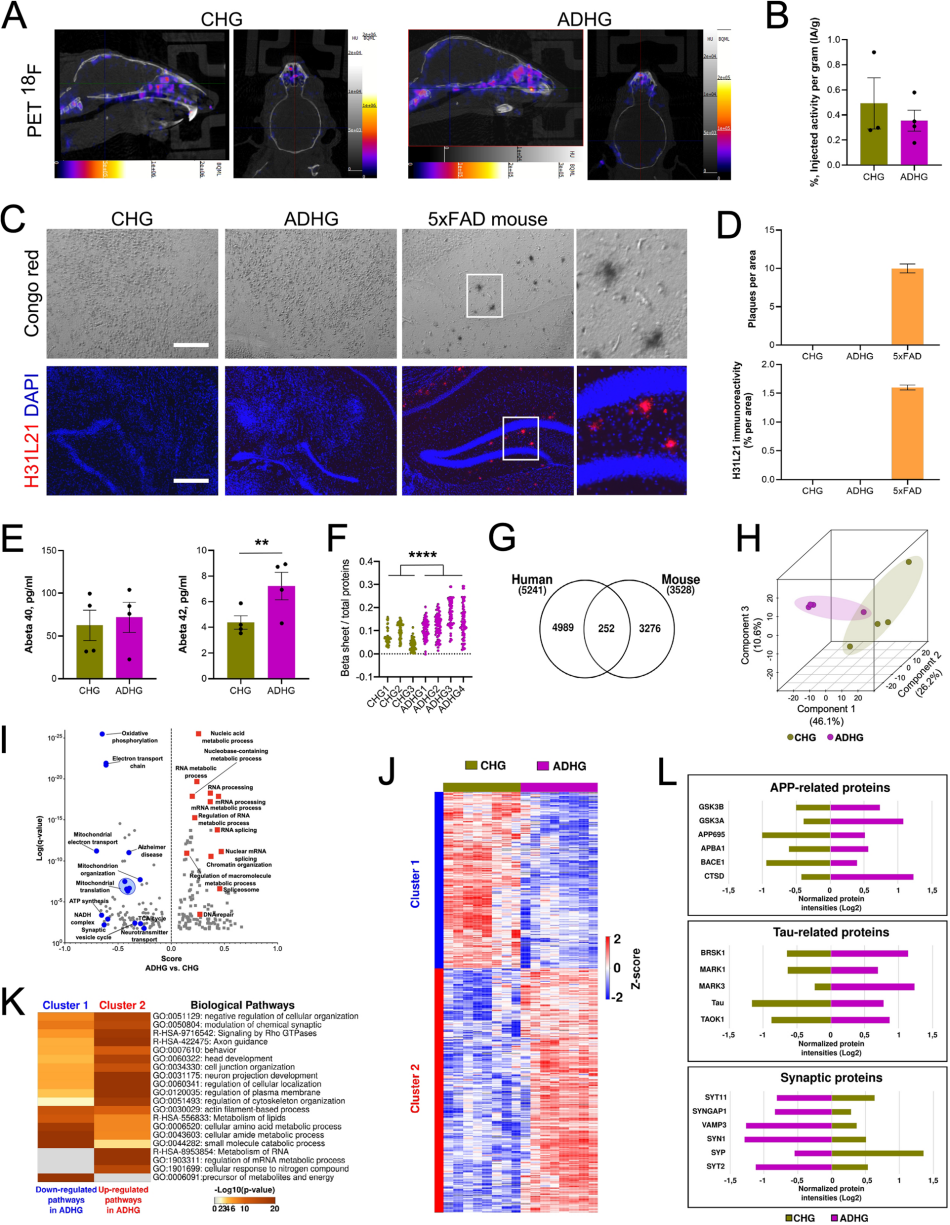
AD human grafts exhibit significant proteomic alterations, but do not show main AD hallmarks **a** and **b** Overview of representative PET/CT images of ^18^F-flutemetamol in control and AD iPSC-derived grafts six months after transplantation into mouse hippocampus. Sagittal and axial views showed no specific uptake related to AD in the brain. Color scale bar shows (from black to white) activity values in PET ^18^F-flutemetamol image. The diagram shows relative uptake values presented as %IA/g. Results are presented as mean ± S.E.M. N = 3–4 animals. **c** and **d** Characterization of Aβ deposition in hippocampi of 6-month-old 5xFAD transgenic mice and control and AD iPSC-derived grafts. Congo Red staining and immunostaining for H31L21 show presence of Aβ deposits only in 5xFAD mice. Scale bars: 200 µm. Results are presented as mean ± S.E.M. N = 4 animals. **e** Accumulation of Aβ measured by MSD in control and AD iPSC-derived human grafts. Results are presented as mean ± S.E.M. N = 4 animals. Statistical analysis by two-tailed t-test. **f** Diagram reflecting the β-sheet structure content in control and AD iPSC-derived human grafts as shown by the absorbance ratios 1,628 cm^−1^, a band characteristic for β-sheet structure, to 1,656 cm.^−1^, a band typically assigned to α-helical or random structures, main components of protein structures used here as a normalization parameter. Results are presented as individual values. n = 42—81 spectra per sample for N = 3–4 animals. P value: **** = P < 0.0001. Statistical analysis by two-tailed t-test. **g** Venn diagram representing the number of identified proteins exclusively derived from AD iPSC-derived human grafts and/or from host mouse brain tissue. N = 4 animals. **h** Principal component analysis using the normalized intensities of proteins quantified in control and AD iPSC-derived human grafts. **i** 1D annotation enrichment analysis of quantified proteins in control and AD iPSC-derived human grafts (Benjamini–Hochberg FDR: 0.02). Underrepresented pathways in ADHG (blue) are mainly enriched by mitochondrial function. Overrepresented biological pathways in ADHG (red) are mostly associated with the dysregulation of RNA metabolism. **j** Hierarchical clustering analysis of dysregulated proteins in AD iPSC-derived human grafts (Two-tailed t-test; *p*-value < 0.05; Benjamini–Hochberg FDR: 0.05) using Pearson correlation distance. The abundance of protein groups decreased and increased is represented in blue and red, respectively. **k** Biological pathways enriched in the protein clusters of the hierarchical clustering analysis of AD iPSC-derived human graft proteome (*p*-value < 0.01). *p*-values are denoted as the –log10 (*p*-value). The grey color symbolizes the absence of that specific biological pathway. **l** Cluster bar representation of significantly altered proteins linked to well-established markers of early molecular dysfunction in AD (Two-tailed t-test; *p*-value < 0.05; Benjamini–Hochberg FDR: 0.05)

The formation of senile plaques caused by the accumulation and aggregation of mainly Aβ42 peptides forming amyloids in the brain extracellular space, is one of the main hallmarks of AD pathology [19]. Positron emission tomography (PET)-mediated imaging of Aβ fibrils in the brain using tracer flutemetamol (^18^F) is very efficient at detecting amyloid deposits in the brain of AD or suspected AD patients [20]. The technique has also been used to define when, during the course of the disease, mouse models of AD develop Aβ plaques [21]. To detect whether Aβ deposition had occurred in 6-month-old ADHG, we subjected the humanized mice to ^18^F-PET imaging. No difference in signal activity was observed in the brain of the animals transplanted with AD hippocampal brain cells compared to those transplanted with control hippocampal brain cells (Fig. 2a, b). This data, which suggested the absence of Aβ plaques in the transplanted animals, prompted us to examine whether amyloid fibrils could be identified in the AD grafts using alternative techniques. In corroboration with the ^18^F-PET imaging data, no staining was identified in either the AD or control graft, after processing of the tissue with either Aβ antibody H31L21 immunohistochemistry or Congo-red staining, compared to AD mouse brain tissue used as positive control (Fig. 2c, d).

Because the human cells that compose the graft are young, it is possible that they exhibit signs of AD cellular pathology prior to amyloid plaque formation. We next utilized MSD multi-array to measure the amount of Aβ40 and Aβ42 peptides present in the human grafts. Levels of Aβ40 peptide were not different between CHG and ADHG. However, levels of Aβ42 peptide were significantly increased in ADHG compared to CHG (Fig. 2e). Additionally, we found increased levels of β-sheet structures measured by Fourier transform infrared (FTIR) micro-spectroscopy. Increased levels of β-sheet structures preceding the formation of amyloid aggregates was previously identified in AD patient iPSC-derived hippocampal spheroids in vitro and in young mouse models of AD [7, 22]. These data which suggested that AD cellular pathology was initiated, prompted us to examine if and what cellular pathways and networks were changed in the AD patient brain cells. Towards this end, we assessed the proteomic landscape in the grafts, by taking advantage of quantitative protein analysis by LC–MS/MS (Additional file 2: Table S1). Using the *Homo sapiens* and *Mus Musculus* databases (Uniprot) integrated into the pipeline of mass spectra searching, we were able to dissect the proteome map derived from the human and mouse cells that composed the grafts. The identification of species-specific unique peptides successfully discriminated the human graft protein profile from the mouse brain proteome, with minimal number of overlapping proteins (Fig. 2g). Principal component analysis (PCA) based on the quantified human global proteome clearly discriminated ADHG from CHG groups (Fig. 2h). Enrichment analysis showed over-representation of RNA metabolic pathways in human cells that composed the ADHG, while biological pathways related to mitochondrial and synaptic function were under-represented (Fig. 2i).

Further analysis allowed us to scrutinize which proteins had altered levels in human cells composing the ADHG compared to CHG (Additional file 3: Table S2). Hierarchical clustering analysis allowed for the identification of two main protein clusters (Fig. 2j). Cluster 1 encompassed proteins whose level was decreased in the human cells composing the ADHG, featured by alterations in energy-related metabolic pathways, and lipid and amino acid metabolism. Increased proteins grouped in the second cluster, characterized by the increment of RNA metabolism, plasma membrane and cytoskeleton regulation, membrane trafficking, and Rho GTPases signaling (Fig. 2k). Since altered APP processing, increased Tau phosphorylation and impaired synaptic transmission play pivotal roles in AD [23], we next focused on several proteins involved in these processes. In addition to observe higher levels of APP and Tau, we found increased levels of proteins involved in APP processing and Tau phosphorylation, coupled to a decrease of several synaptic proteins in human cells composing the ADHG, compared to CHG (Fig. 2l).

Taken together, these data suggest that important pathways and networks are altered in human cells in the ADHG; and that they coincide in time with the formation of β-sheet structures, increase Aβ42, and accumulation of APP and Tau, but precede the formation of senile Aβ plaques.

### Comparison of proteomic changes across human AD hippocampal spheroids, human cells in ADHG and AD patient post-mortem hippocampal tissue suggests a transition of cellular changes from early to end-stage AD cellular pathology

**Fig. 3:**
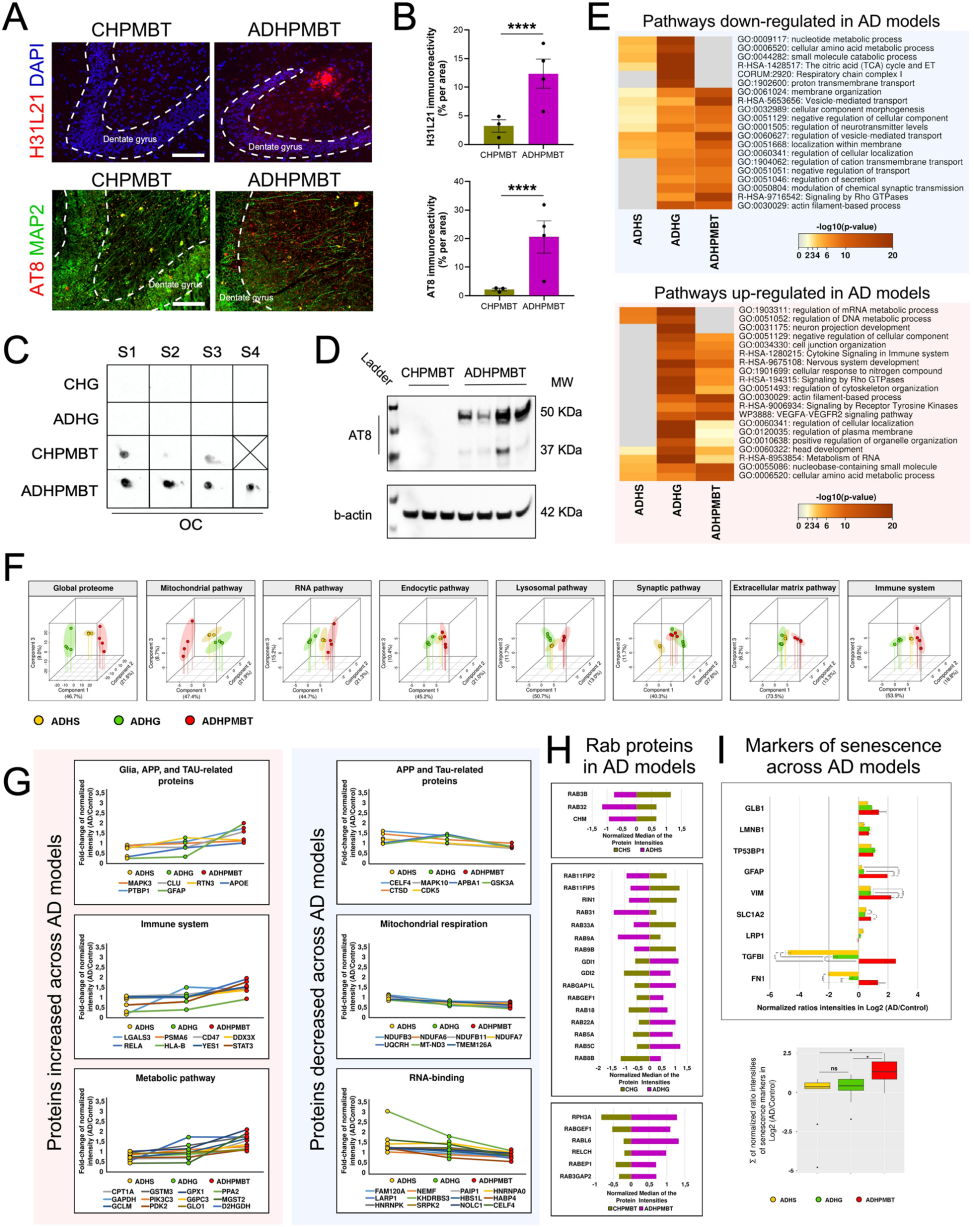
Label-free quantitative proteomics reveals common and distinctive alterations in iPSC-based AD models and postmortem hippocampi from AD patients **a** and **b** Characterization of amyloid-β deposition and phosphorylation of tau protein in postmortem hippocampi of AD patients and non-demented controls. Immunostaining for H31L21 show presence of Aβ deposits in postmortem hippocampi of AD patients. H31L21 immuno-positive area was quantified relative to the total area. Immunostaining for AT8 show increased tau phosphorylation in postmortem hippocampi of AD patients. AT8 immuno-positive area was quantified relative to the total area. Results are presented as mean ± S.E.M. N = 3–4 animals. P value: **** p < 0.0001. Statistical analysis by t-test. CHPMBT: control human postmortem brain tissue; ADHPMBT: AD human postmortem brain tissue. **c** Dot blot immunoassay performed using OC antibody specific to amyloid fibrils showing their presence in postmortem hippocampi of AD patients and non-demented controls, but not in human grafts (b). Scale bars: 200 µm. **d** Western blotting analysis of phosphorylation of tau protein with actin blot included as a loading control. N = 3–4 human samples. **e** Comparison of the biological pathways dysregulated in AD iPSC-derived human spheroids (ADHS), AD iPSC-derived human grafts, and AD human postmortem brain tissues with respect to the control conditions (*p*-value < 0.01). *p*-values are denoted as the –log10 (*p*-value). The grey color symbolizes the absence of that specific biological pathway. **f** Principal component analysis of the normalized protein intensities that participate in key signaling pathways affected in AD. The proteomic profiles of each biological pathway are unique for each AD iPSC-derived model and PMBT, showing a clear separation between them. **g** Selected proteins significantly altered across the different AD models. Median fold-change (AD/Control) of normalized intensities; One-way ANOVA *p*-value < 0.05; Benjamini–Hochberg FDR < 0.05); Tukey HSD FDR < 0.05. **h** Quantification of dysregulated Rab proteins in each AD model. Bar graphs show the median of normalized protein intensities. Statistical significance was assessed by a two-tailed t-test; *p*-value < 0.05; Benjamini–Hochberg FDR < 0.05 and the log2 fold-change cutoffs of ± 0.5. **i** Quantification of dysregulated markers of senescence across the different AD models. One-way ANOVA *p*-value < 0.05; Benjamini–Hochberg FDR < 0.05; Tukey HSD FDR < 0.05

To better understand which disease stage was modelled within the human grafted AD cells, we compared the proteomic changes taking place in human cells in ADHG with those in APP variant hippocampal spheroids (ADHS) grown for 100 days in vitro [7] and human AD hippocampal postmortem brain tissue (ADHPMBT; Additional file 4: Table S3), representing early and end stage AD, respectively. First, we confirmed the presence of amyloid plaques in ADHPMBT sections by immunohistochemistry and dot-blot assay, the latter using OC antibody that specifically recognizes amyloid fibrils [24] (Fig. 3a–c). Additionally, immunohistochemistry and Western blotting performed with AT8 antibody showed increased phosphorylation of Tau in ADHPMBT compared to control tissue obtained from non-demented healthy controls (Fig. 3a, b and d).

Next, we reasoned that the presence of amyloid deposition and hyperphosphorylated Tau in ADHPMBT seen in end stage AD, should be paralleled by proteomic alterations in line but more advanced than those identified in the ADHG and ADHS. To validate this hypothesis, we mapped the proteome in the two iPSC-based models and ADHPMBT and examined the main commonly dysregulated protein pathways (Fig. 3e). Pathways linked to DNA and mRNA metabolism were increased in the two iPSC-based models of AD, but not in ADHPMBT. In contrast, cytokine signaling, membrane trafficking, and cytoskeleton organization were upregulated in the human cells composing the ADHG and ADHPMBT, but not in the ADHS. Mitochondrial metabolism imbalance was shared between ADHS and human cells composing the ADHG, while decrease in transport regulation and neurotransmission was shared between human cells composing the ADHG and ADHPMBT (Fig. 3e).

The acquisition of the protein profiles in the iPSC-based models and ADHPMBT allowed us to further interrogate if they shared proteomic alterations. The common total proteins quantified in iPSC-based models and ADHPMBT depicted distinctive and unique signatures of dysregulated biological pathways. These were not only identifiable at the global level, but also when analysing each pathway separately (Fig. 3f). To compare the relative abundance of AD-related proteins across the three AD systems, we expressed their amount as a fold change AD/control (Additional file 5: Table S4). The fold change of glial fibrillary acidic protein (GFAP), an astrocytic cytoskeletal protein, known to be markedly upregulated in brains of AD patients [25], increased from ADHS and ADHG to ADHPMBT. In line with this, the fold change of signal transducer and activator of transcription 3 (STAT3), a transcription factor regulating many pathways associated with astrogliosis [26, 27] was elevated from ADHS to ADHPMBT. Similarly, the fold change of several proteins involved in APP processing, Tau phosphorylation, immune system activation, and metabolic-related proteins was minimal in ADHS and maximal in ADHPMBT, reflecting progression of AD cellular pathology from AD iPSC-based models to postmortem ADHPMBT (Fig. 3g). In contrast, the fold change of proteins involved in mitochondrial respiration and RNA-binding, as well as other proteins related to APP and Tau decreased from ADHS to ADHPMBT. These data likely reflected events that occur early in AD cellular pathogenesis (Fig. 3g).

Recently, human iPSC-derived neurons whose genome was edited to express different genes leading to familial AD, revealed endosomal dysfunction as a shared disease-associated cellular phenotype [11]. In line with this work, we examined the levels of several members of the Rab superfamily, small GTPase proteins containing many key regulators of endosome trafficking and remodelling [28], in all three AD systems. While only three Rab proteins were dysregulated in ADHS, 16 members of the Rab superfamily were altered in ADHG and 6 in ADHPMBT. Among them, RABGEF1, a guanine nucleotide exchange factor for RAB5 [29], a key regulator of endosome fusion and trafficking [11, 30], was elevated in both ADHG and ADHPMBT. Interestingly, two isoforms of RAB5, RAB5A and RAB5C were increased only in ADHG. Finally, to evaluate the advance of cellular pathogenesis in the graft, we analyzed the abundance of several senescence markers across all three AD systems. Because proteomics of the samples was not performed at the same time, we expressed their amount as a fold change (AD/control). Data analysis showed prevalence of senescence-related proteins in ADHPMBT compared to ADHG and ADHS, and their mild increase in ADHG compared to ADHS (Fig. 3i).

Because significant protein alterations are shared between human cells in the ADHG and ADHPMBT, and senile plaques are absent in ADHG, our data suggests that the cellular pathology within human cells in the ADHG may be reminiscent of the early/prodromal stage.

### Dysfunction of cellular biological pathways identified in host brain cells

**Fig. 4:**
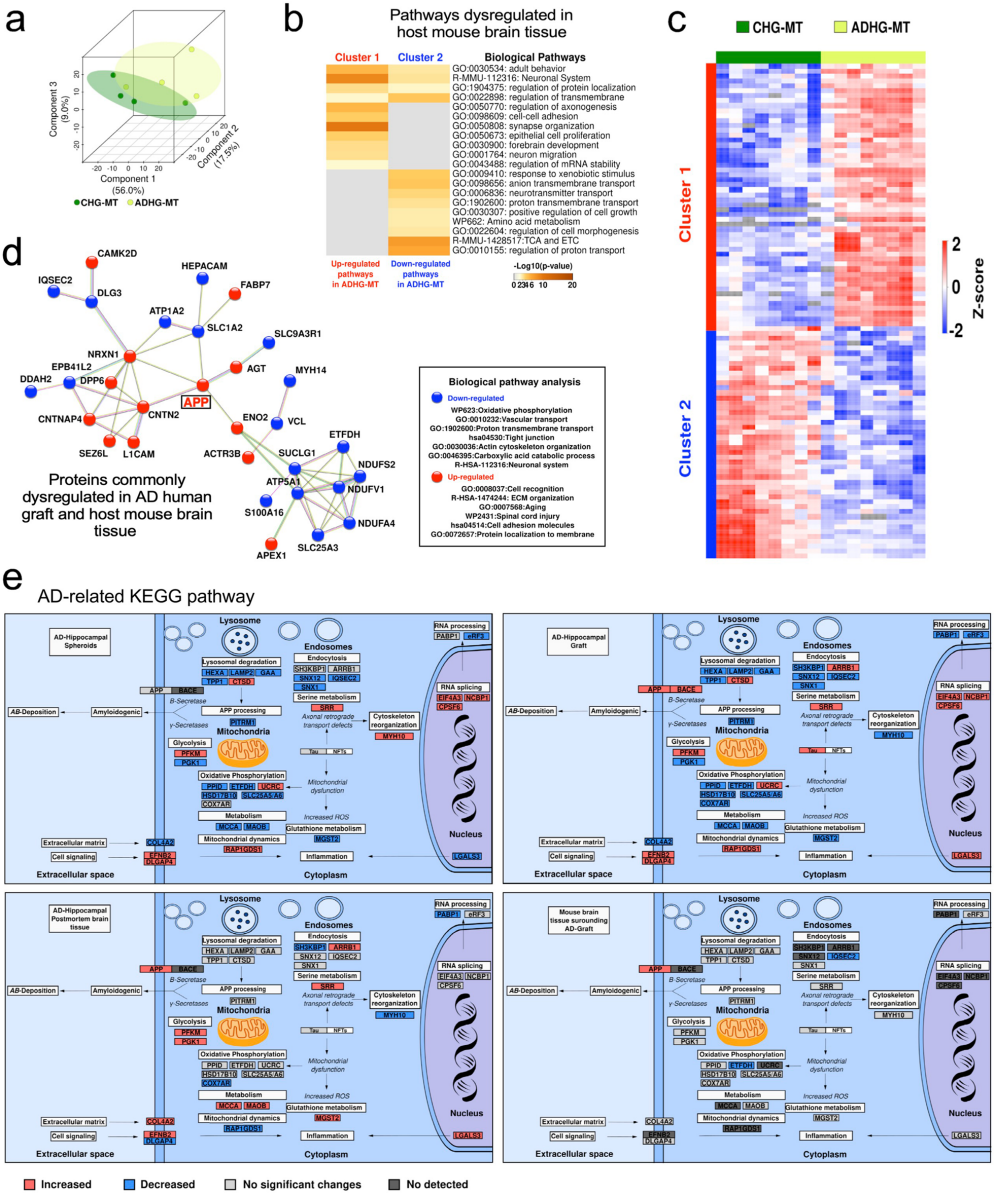
Label-free quantitative proteomics reveals alterations in the host mouse brain tissue induced by AD human grafts **a** Principal component analysis of murine proteins identified in control and AD-grafted mouse hippocampi. CHG-MT (control human graft-mouse tissue); ADHG-MT (AD human graft-mouse tissue). **b** Hierarchical clustering analysis of dysregulated host mouse proteins (Two-tailed *t*-test; *p*-value < 0.05; Benjamini–Hochberg FDR < 0.05) using Pearson correlation distance. The abundance of protein groups decreased and increased is represented in blue and red, respectively. **c** Pathway analysis of the protein clusters in C (*p*-value < 0.01). *p*-values are denoted as the –log10 (*p*-value). The grey color symbolizes the absence of that specific biological pathway. **d** Interaction network and pathway enrichment of the commonly dysregulated proteins between AD iPSC-derived human grafts and the host mouse tissues (Two-tailed t-test; *p*-value:0.05; Benjamini–Hochberg FDR:0.05). Decreased and increased proteins are represented in blue and red, respectively. **e** Mapping of the dysregulated proteins participating in the AD pathway reported in the KEGG database. The analysis was centered on the dysregulated proteins (AD vs. Control) in the ADHG model in comparison to ADHS, ADHPMBT, and ADHG-MT. Protein groups with decreased and increased abundances are represented in blue and red, respectively. Proteins with no significant changes in their abundance and not detected are represented in grayscale

We next asked whether the grafted human APP mutant cells could potentiate cellular pathways and network alterations in surrounding mouse cells. Proteomic analysis allowed us to discriminate 3276 mouse host proteins (Fig. 2g). The PCA analysis clearly showed separation of the proteome in the mouse tissue adjacent to the AD human graft (ADHG-MT), compared to control (CHG-MT) (Fig. 4a; Additional file 6: Table S5). Clustering analysis identified two main groups of dysregulated pathways in the ADHG-MT (Fig. 4b, c). Cluster 1 exhibited upregulated pathways involved in regulating mRNA stability, transmembrane regulation, and synapse organization, whereas cluster 2 showed downregulated pathways mainly linked to metabolism, such as the citric acid cycle and electron transport chain (Fig. 4b, c).

We next examined how proteins whose levels were changed in both human and mouse cells connected with each other. We used the UniProt tool to convert the *Mus musculus* entries to *Homo sapiens*. The data analysis demonstrated an overlap in crucial AD-related proteins and pathways (Fig. 4d). Notably, there was an increase of proteins related to cell adhesion, extracellular matrix organization, and membrane transport. Simultaneously, we detected a decrease in the number of proteins involved in oxidative phosphorylation, ion transport, and cytoskeleton organization (Fig. 4d). The most surprising finding was the identification of increased APP levels in both human grafted cells and mouse host cells.

Considering these findings, we decided to compare the abundance of proteins involved in the AD-related KEGG pathway (hsa05010) across the four systems identified in our study, to map out the cellular origin and progression of AD pathogenesis: ADHS, human cells in the ADHG, ADHPMBT and ADHG-MT (Fig. 4e). Several proteins involved in lysosomal degradation, endocytosis, APP processing, oxidative phosphorylation, serine and glutathione metabolism, RNA processing and splicing were commonly dysregulated in the 4 systems. At the same time, proteins involved in endocytosis, serine metabolism, cytoskeleton reorganization, and oxidative phosphorylation were commonly dysregulated in human cells in the ADHG and ADHPMBT. In addition, the mitochondrial protein ETFDH involved in oxidative phosphorylation and the postsynaptic protein IQSEC2 involved in endocytosis, were commonly decreased in ADHS, human cells in the ADHG and ADHG-MT. Importantly, we identified APP as a protein commonly increased in human cells in ADHG, ADHPMBT and ADHG-MT (Fig. 4e).

Altogether, the data suggest that different mechanisms are at play at different stages of AD pathology represented by the 4 systems, and that APP is central to AD cellular pathogenesis.

## Discussion

Most AD cases have an idiopathic origin. Transgenic AD animals have been the most common models to develop therapeutic strategies. Yet, animal models cannot inform on the cellular origin and progression of AD patient brain cell dysfunction, despite exhibiting some degree of similarities in differentially expressed gene signatures that partially recapitulates AD patient molecular subtypes [31]. Because human iPSCs harbor the genetic background of the individual they are derived from, they may serve as a better alternative to understand AD cellular pathogenesis and develop treatments [32]. For example, work employing patient iPSCs has showed that haplotypes are important modulators of genetic risk factors for late-onset AD [33]. Recently, we and others successfully utilized AD patient iPSCs to generate 2D neuronal cell cultures and more complex free-floating organoid or spheroid structures, to uncover AD cellular phenotypes and test therapeutic strategies [5, 7, 12, 33–35]. These studies, together with those that generated RNA sequencing data from AD postmortem brain tissue [31, 36, 37], have highlighted cell type specific dysfunctions and heterogeneity of patient cellular phenotypes. However, when grown in vitro, iPSC-derived brain cells lack the microenvironment that exists in the brain, preventing a more patient-like pathophysiological analysis of cellular phenotypes. To overcome this limitation several studies contemplated the possibility to study human cellular pathogenesis in humanized models. The human-rodent chimeric models allowed for the study of the effect of the AD brain microenvironment on human neurons [17] and glia [18].

Here, we thought to examine AD cell autonomous dysfunction in the healthy brain microenvironment, that naturally provides support to neurons and waste clearance, and to study the effect of the AD grafted brain cells on surrounding non-diseased host cells. We first demonstrated that the human grafted cells survived in the host mouse brain. Using LC–MS/MS, we observed increased abundance of APP and Tau, and the proteins involved in their processing, in ADHG compared to control. The proteomic changes were coupled to increased β-sheet structures and Aβ42 levels. Yet, senile plaques measured by Congo red and amyloid antibody H31L21 staining were absent. We speculate that this could be attributed to the highly functional waste clearance system of the healthy host brain [38]. Another possibility lies in the fact that cell transplantation was performed in young mice aged 6–8 weeks. Najm and colleagues recently showed Aβ aggregation in iPSC-derived cortical neurons carrying ApoE4 risk factor, 7 months post-transplantation into the hippocampus of mice grafted when aged 6 months [16]. Alternatively, senile plaque formation may require longer period to form for the gene variant we studied. Indeed, plaques start to develop in the Thy1-APPLon mice when aged 10 months [39]. These points should be addressed in future studies and include the analysis of cellular phenotypes over longer periods both in vitro and in vivo, and with grafted cells having different genetic backgrounds.

Human astrocytes have emerged as a major player in AD [34, 40–43]. RNA sequencing data clearly revealed pathways and network dysfunction in astrocytes of the AD postmortem brain [44–46]. Not only these cells can become reactive when exposed to Aβ in vivo [18], and their secretion of cytokines enhanced following stimulation [34], their ability to uptake glutamate, release lactate and propagate calcium is altered if carrying a genetic variant leading to early onset AD [34]. We previously observed similar features when Parkinsonian and control iPSC-derived astrocytes were exposed to alpha-synuclein protein aggregates [47]. Here, ADHG contained fewer human astrocytes that CHG. However, ADHG contained many more host astrocytes. Possibly, the ADHG had attracted and activated a greater number of host astrocytes. Future work should explore whether cell autonomous dysfunctions exist in APP p.V717I astrocytes, in addition to the increase in β-sheet structure formation previously identified [7].

Here, the grafts were composed of both human and mouse cells. Thus, we had to conduct a database search of the LC–MS/MS spectra against the combined *Homo sapiens* and *Mus musculus* databases (Uniprot) to quantify species-specific unique peptides and investigate them individually. Proteomic analysis revealed profound cellular alterations in ADHG. Mitochondria homeostasis is one of the earliest impaired intracellular processes in AD [48–50]. In line with this, we identified several mitochondria associated proteins whose levels were changed in ADHG. Importantly, we observed an increase in protein kinases and proteases, including but not limited to GSK3B, GSK3A, BACE1, MARK1 and MARK3. These proteins, over-activity of which accounts for memory impairment, tau-hyperphosphorylation and increased Aβ production [51] increase with age and in AD [52–54]. Additionally, we observed an important decrease in several synaptic proteins in the ADHG, which is in line with the observations that synaptic pathology is an early feature of AD [55, 56].

Like many neurodegenerative diseases, AD is considered a “prion-like” disease [57–59]. Evidence suggests that both Aβ and Tau protein aggregates can spread throughout the brain in “prion-like” ways to trigger cellular dysfunction and seed de novo aggregation in recipient cells [57, 60–62]. In line with this, secretomes of AD iPSC-derived neurons delivered to the adult rat hippocampus resulted in impaired synaptic plasticity via a common pathway that is mediated by cellular prion proteins [59]. Interestingly, we identified several common proteomic alterations in both human and mouse proteomes, suggesting that cellular AD pathology within transplanted human AD hippocampal cells induced alterations in the mouse host cells proteome. One of the commonly dysregulated proteins was APP, accumulation of which occurs early in the cascade of events that results in senile plaque formation in AD brain. Interestingly, not only pathogenic variations in APP but also higher levels of wildtype APP either due to chromosomal translocation (trisomy 21) or APP gene locus duplication, lead to early onset AD [63–65]. We speculate that increased APP levels may be induced by Aβ peptides secreted by the human AD cells, which would be in line with earlier reports [66]. While future studies may elucidate the mechanism leading to increased APP level in host cells, current effort should aim at developing strategies to lower APP level which may be a relevant therapeutic avenue to slow down AD progression.

### Limitations of this study

We acknowledge several limitations in our study. Recent single cell/nuclei sequencing data clearly indicate that genes whose variants lead to early onset AD or genetic risk factors for late onset AD, are prominently expressed in glia in the human brain [36, 44–46, 67]. For instance, the expression of Presenilin 1 and APP genes is higher in oligodendrocytes than neurons (https://www.brainrnaseq.org/). Moreover, a study by Wang and colleagues identified well established pathways implicated in AD in oligodendrocytes while characterizing molecular networks associated with AD from 1053 postmortem brain samples across 19 cortical regions of 125 individuals with a severity spectrum of dementia and neuropathology of AD [36]. This suggests that oligodendrocyte dysfunction could play an important role in AD pathogenesis, for example due to impaired myelination via cholesterol dysregulation [68]. Indeed, white matter hyperintensities have been found elevated among individuals with early onset AD up to 20 years before the expected onset of symptoms [69]. Despite our capability to generate all brain cell types from human iPSCs [7, 47, 70], we have not yet developed a protocol to generate highly regionalized hippocampal spheroids containing neurons, astrocytes, and oligodendrocytes. Such a model system could allow for the examination of myelination defects and demyelination, both in vitro and in vivo. Likewise, microglia, cells of mesodermal/mesenchymal origin, are important in the formation of senile plaques [71]. TREM2 pathogenic variants supposedly lead to AD via decreased Aβ clearance due to defective phagocytosis by microglia [72]. The incorporation of iPSC-derived microglia could provide further information into neuronal phenotypes [73].

Some animals experienced epileptic seizures; for ethical reasons, these had to be put down, which is also the reason why we ended our study 6 months post-transplantation and had a reduced number of animals per group. We speculate seizures to be consequent to dysregulation of local hippocampal networks induced by the graft over time. In future studies, we could transplant either fewer brain cells or purified non-dividing cell types and follow how cellular pathology would develop in them over longer periods. Alternatively, we could graft whole brain spheroids by cranial window surgery, as performed for organoids [74, 75] and providing these would not increase in size over time. This would allow us to perform two-photon imaging studies to reveal neuronal network activity of the AD patient transplanted cells.

Here, we used a sex- and age-matched control iPSC line, rather than an isogenic iPSC control line. The control line was used in several of our studies and showed no disease phenotype compared to iPSC lines generated from patients with neurodegenerative diseases [7, 76]. Mutation-corrected isogenic control lines are often preferred when studying familial forms of diseases. Although these cannot be generated from idiopathic forms which represent 95% of all AD cases, their use can help minimize experimental variability caused by donor differences.

Finally, the hippocampal tissues and iPSC-derived brain cells analyzed originated from different patients. Whereas iPSC-derived brain cells carried the APP London mutation, the post-mortem brain tissue samples originated from idiopathic AD cases. Recently, Lagomarsino and colleagues generated induced cortical neurons from iPSC lines obtained from a large cohort of deeply phenotyped individuals, including sixteen neuropathologically diagnosed with AD and Alzheimer’s dementia [77]. RNA sequencing and proteomic data revealed shared pathways and network dysfunction for iPSC-derived induced neurons and postmortem brain cells of the same individuals. Nevertheless, it could be argued that since all AD cellular pathways and network dysfunction converge towards the same cellular hallmarks, though there exists different molecular signature subtypes between AD patients [31], the tissue analyzed represented the best available resource for the study. This issue could be circumvented if APP London mutation brains become available.

## Conclusions

This study highlights the promise of studying patient cellular pathogenesis in vivo, in experimental humanized rodent models. By transplanting iPSC-derived brain cells into the mouse brain, we were able to demonstrate what cellular pathways and networks are altered in young AD patient hippocampal cells and compare them to those present in ADHS grown in vitro and patient post-mortem tissue. Importantly, our data indicate that the AD grafted cells had induced pathways and network changes within the mouse host cells. Future studies may help determine if cellular pathology had transferred from the AD grafted cells to host cells via a “prion-like” mechanism [78–80]. This new human-mouse chimeric model complements existing recourses [77, 81], and represents a powerful tool to better study the mechanisms underlying the cellular origins and progression of human AD pathogenesis, in vivo. We anticipate the future use of humanized rodent models to develop and test therapeutic strategies for AD, and importantly, validate target engagement in human brain cells in vivo, prior to moving experimental therapies to clinical trials.

## Experimental procedures

All materials were purchased from ThermoFisher Scientific, unless specified.

### Human induced pluripotent stem cell lines

The generation and characterization of human iPSC lines CSC-37N (healthy control aged 56-year-old) and CSC-17F (APP p.V717I, heterozygous, aged 50-year-old) has been previously reported [7].

### Generation of hippocampal spheroids

After several passages in vitro, iPSCs were differentiated into hippocampal spheroids as previously described [7]. Briefly, to form EBs, iPSC colonies were dissociated using dispase (1 mg/ml) and transferred into ultra-low adherent flasks (Corning) in WiCell medium supplemented with 20 ng/ml FGF2 and 20μM ROCK-Inhibitor Y-27632 (Selleck Chemicals, Munich, Germany). Next day, WiCell was replaced with neural induction medium (NIM) composed of advanced DMEM/F12, 2% B27 Supplement without vitamin A (v/v), 1% N2 Supplement (v/v), 1% NEAA (v/v), 2 mM L-glutamine and 1% Penicillin–Streptomycin (v/v) supplemented with LDN-193189 (Stemgent, 0.1 μM), Cyclopamine (Selleck Chemicals, 1 μM), SB431542 (Sigma-Aldrich, 10 μM) and XAV-939 (Tocris, 5 μM). On the tenth day, the free-floating spheres were transferred to neuronal differentiation medium (NDM) containing Neurobasal® medium, 1% N2 (v/v), 1% NEAA (v/v), L-glutamine and 1% Penicillin–Streptomycin (v/v) supplemented with CHIR-99021 (Stemgent, 0.5 µM) and brain derived neurotrophic factor (BDNF, PeproTech, 20 ng/ml). On the day in vitro 50, spheroids were dissociated into single cells with Trypsin 1X and resuspended in PBS to a final concentration of 100 000 cells/μl for transplantation into the mouse brain.

### In vivo* transplantation*

For in vivo transplantation, male 6–8 weeks old RAG-2-deficient mice (Janvier Labs, France) were used. The mice were anesthetized with isoflurane (Baxter, Deerfield, IL) in oxygen (initial dose of 5% which was reduced to 1–1.5% for maintenance of surgical depth anesthesia) delivered through a nose mask during the surgery. The body temperature was maintained at 37.0°C during the surgery using a thermostatically controlled rectal probe connected to a homeothermic blanket. The skull was exposed by the skin incision after subcutaneous analgesia with Marcaine (50 μl of 2.5 mg/ml stock solution, Astra Zeneca). Transplantation was stereotaxically performed for each mouse bilaterally through drilled holes in the skull using a 5-μl Hamilton syringe with 32-gauge needle and an injecting minipump (Nanomite Injector Syringe Pump; Harvard Apparatus, Holliston, MA, USA). A volume of 2 μl of cell suspension was injected at a rate of 0.5 μl/min at the following coordinates (from bregma and brain surface): anterior/posterior (AP): -2.0 mm; medial/lateral (M/L): ± 1.5 mm; dorsal/ventral (D/V): -1.7 mm. The needle was left in situ for 7 min after injection before being slowly raised, and the wound was sutured.

### PET imaging

Six months after transplantation, the mice were prepared for imaging in a small animal PET/CT scanner (nanoScan PET/CT, Mediso, Hungary). Eight mice (4 controls and 4 AD) were injected with a mean activity of 14.7 MBq (range: 4.6–25.2 MBq) of 18F-flutemetamol intravenously in a tail vein. 70 min after injection of the radiopharmaceutical, a PET scan was performed with a scan time of 20 min. Prior to and during scanning, the mice were anaesthetized with isoflurane and kept warm via a closed flowing air system in the animal beds. The respiration of the animals was monitored during the whole scan. After the PET scan and sequentially without moving the animal between scans, a 15 min CT scan was performed at 45kV and a 900 ms exposure time. PET image reconstruction was performed using the Tera-Tomo™ three-dimensional (3D) PET image reconstruction engine with 4 iterations and 6 subsets and a voxel size of 0.4 × 0.4x0.4 mm3 (Mediso, Hungary). The CT image was used for attenuation correction and as anatomical reference during analysis. One of the control animals moved during the PET scan, and thus had to be excluded from the analysis. Image analysis was performed using the software Vivoquant™ 4.0patch1 (InviCRO, Needham, MA, USA). Region of interests (ROIs) were drawn in the fused PET/CT images and values of activity uptake and volume were extracted for calculation of %IA/g for each animal.

### Rodent brain tissue collection and processing

Six months after transplantation, the mice were transcardially perfused with PBS. The brain tissues containing grafts from the left and right brain hemisphere were dissected, cut into two halves each, snap frozen on liquid nitrogen and stored at -80°C for further biochemical analysis. For immunohistochemistry, the mice were transcardially perfused with PBS followed by perfusion with 50 ml of 4% paraformaldehyde (PFA). The dissected brains were post-fixed in the same fixative overnight at 4°C. After fixation, brains were cryoprotected in 30% sucrose (Sigma- Aldrich) for 48 h at 4°C before being cut into 30 μm thick coronal serial sections on a sliding microtome (Leica Biosystems, Wetzlar Germany). The free-floating brain sections were then stored in antifreeze solution at −20°C until immunohistochemical staining.

### Human postmortem brain tissue processing

The human postmortem brain samples, hippocampi from AD patients and non-demented controls (Additional file 7: Table S6) were obtained from The Netherlands Brain Bank, Netherlands Institute for Neuroscience, Amsterdam (open access: www.brainbank.nl). All Material has been collected from donors for or from whom a written informed consent for a brain autopsy and the use of the material and clinical information for research purposes has been obtained by the NBB. The frozen hippocampi samples were cut into 20 μm thick sections on a cryostat (Leica Biosystems) and then stored at -80°C until immunohistochemical staining or biochemical analysis.

### Immunostainings, microscopy, and image analyses

For immunohistochemistry, frozen mouse or human sections were air-dried for 1 h, washed 3 times in PBS, blocked for 1h at RT with 5% normal donkey serum (NDS, VWR) in PBS with 0.25% Triton-X (Sigma-Aldrich) and incubated overnight with target primary antibodies (Additional file 8: Table S7) prepared in 5% NDS in PBS with 0.25% Triton-X at 4ºC. Prior to blocking, the human sections were fixed with 4% PFA for 1h at RT. On the next day, the sections were incubated with appropriate Alexa-fluor 488 or 555- conjugated secondary antibodies (Additional file 8: Table 7) in PBS for 2h at RT in the dark. Cell nuclei were counterstained with DAPI (1:10,000). Congo Red staining was performed using Congo Red Staining Kit (Merck) following the manufacturer's instructions. Image acquisition was performed using inverted epifluorescence microscope LRI-Olympus IX-73 equipped with fluorescence filters. Automated quantitative image analysis was performed with the ImageJ software or MetaMorph Software V7.6 (Molecular Devices) using the Multi-Wavelength Cell Scoring application. For a specific marker, positive cells were identified as having signal intensity above the selected intensity threshold. Intensity thresholds were set blinded to sample identity.

### MSD MULTI-ARRAY Assay

Aβ40 and Aβ42 levels were quantified in brain lysates using the multiplex Aβ Peptide Panel 1 kit (6E10, MesoScale Discovery, USA) according to the manufacturer’s protocol.

### Western blot analysis

For WB, samples were mixed with loading buffer, heated at 70 °C for 10 min and loaded into Bolt 4–12% Bis–Tris Plus gels. After electrophoresis the samples were transferred to polyvinylidine difluoride (PVDF) membranes. Membranes were blocked in PBS containing 0.1% Tween-20 (PBST) and 5% milk or 5% BSA and incubated with target primary antibodies (Additional file 9: Table 8), overnight. Next day, membranes were incubated with HRP-conjugated secondary antibodies (Additional file 9: Table S8) for 1 h diluted in PBST and 5% milk or 5% BSA. The blots were then visualized using Pierce Enhanced Chemo-Luminescence solution and imaged in a Biorad Chemi-Doc chemo-luminescence system (BioRad).

#### Dot blot analysis

For Dot blot analysis, 20 ug of proteins of each sample was deposited directly onto PVDF membranes. The blots were incubated with OC antibody (Sigma-Aldrich, Sweden, #AB 2286, 1:5000) that targets amyloid fibrils (Kayed et al., 2007), overnight. The next day, the membranes were washed and incubated with an anti-mouse HRP-conjugated secondary antibody (R&D Systems; 1:1000) for 1 h. The blots were then visualized using Pierce Enhanced Chemo-Luminescence solution and imaged in a Biorad Chemi-Doc chemo-luminescence system (BioRad).

#### Fourier transformed infrared micro-spectroscopy

For FTIR micro-spectroscopy analyses, fresh frozen mouse brain tissues containing human grafts were cut into 16 μm thick sections on a cryostat. Sections were mounted on the 1 mm thick CaF2 round 10 mm spectrophotometric windows. Infrared spectra were taken from RANDOM areas of the section at the SMIS beamline of the SOLEIL synchrotron (SMIS beamline, France) using a Thermo Fisher Scientific Continuum XL FTIR microscope through a 32 × magnification, 0.65 NA Schwarzschild objective. For the collection, parameters were at spectral range 1000–4000 cm^−1^, in transmission mode at 4 cm^−1^ spectral resolution, with 10 µm × 10 µm aperture dimensions, using 256 codded scans. Background spectra were collected from a clean area of the same CaF2 window. All measurements were made at room temperature. For analysis of FTIR spectra OPUS software (Bruker) and Orange (University of Ljubljana) were used and included atmospheric compensation. Derivation of the spectra to the second order using Savitsky-Golay of 3^rd^ polynomial order 3 with 9 smoothing points, was used to unmask the number of discriminative features and to eliminate a contribution of a baseline.

#### Protein extraction and determination

Brain tissues for immunoassays were resuspended in 200 µl of ice-cooled lysis buffer (50 mM Tris-buffer, 150 mM NaCl, 0.05% Tween-20, protease, and phosphatase inhibitor cocktail (1X)). Then, the proteins were extracted by sonication executing 40 cycles of 15 s on and 15 s off at 4 °C using a Bioruptor plus (model UCD-300, Diagenode). After lysis, samples were centrifuged at 4 °C, 20.000 g for 20 min, supernatants were collected and stored at −80 °C until further use. Total protein amount was determined using the Pierce™ BCA Protein Assay Kit according to the manufacturer instructions. Protein extraction of the hippocampal spheroids for proteomic analysis was performed as previously described (Pomeshchik Y et al., 2020). Proteins from the hippocampal graft and hippocampal post-mortem brain tissues were extracted using a lysis buffer of 25 mM DTT, 10 w/v% SDS in 100 mM Triethylammonium bicarbonate (TEAB). Samples were sonicated using 40 cycles of 15 s on/off at 4 °C in the Bioruptor plus (model UCD-300, Diagenode) after boiling at 99 °C for 5 min. Samples were centrifuged at 20,000 g for 15 min at 18 °C, and the supernatant was collected. Protein concentrations were measured using the Pierce™ 660 nm Protein Assay with ionic detergent compatibility reagent (Thermo) and preserved at −80 °C until further use.

#### Protein digestion for proteomic analysis

Samples were alkylated with 50 mM IAA for 30 min in the dark at room temperature. Protein digestion was performed using the S-Trap™ 96-well plate following the manufacturer´ instructions (PROTIFI. S-Trap™ 96-well plate digestion protocol. https://files.protifi.com/protocols/s-trap-96-well-plate-long-1-4.pdf.) Briefly, 95μL of 50 mM TEAB containing LysC (enzyme: substrate,1:50) was added to each sample and incubated for 2 h at 37 °C, followed by the addition of 30 µL of 50 mM TEAB containing Trypsin (enzyme: substrate,1:50). Samples were incubated overnight, at 37 °C. The reaction was stopped by acidifying the samples with 40 µL of formic acid (FA). Peptides were dried in a speed-vac and resuspended in 0.1% trifluoroacetic acid (TFA)/2%acetonitrile (ACN). Peptide concentrations were measured using the Pierce™ Quantitative Colorimetric Peptide Assay.

#### LC–MS/MS analysis and database searching

Two micrograms of peptides were separated on an Ultimate 3000 RSCLnano pump system using an Acclaim PepMap100 C18 (5 μm, 100 Å, 75 μm i.d. × 2 cm, nanoViper) trap column and an EASY-spray RSLC C18 (2 μm, 100 Å, 75 μm i.d. × 50 cm) analytical column coupled to a Q Exactive HF-X mass spectrometer. The flow rate was set to 300 nl/min for 120 min, with a column temperature of 60°C. For the chromatographic gradient 0.1% FA as solvent A and 80% ACN/ 0.1%FA as solvent B were used. Mass spectra were acquired using the data-dependent acquisition mode. Full scans were collected at 120,000 resolution with a maximum injection time (IT) of 100 ms and a target AGC value of 3e06. The 20 most intense ions were selected for fragmentation using a normalized collision energy (NCE) of 28. MS2 scans were acquired with a resolution of 15,000, a target AGC value of 1e05, and a maximum IT of 50 ms.

The data were searched against the UniProt human database using the SEQUEST HT algorithm in the Proteome Discoverer (PD) 2.3 software. The hippocampal graft samples were additionally searched against a combined *Homo sapiens* and *Mus musculus* database (UniProt). Cysteine carbamidomethylation was considered as static modification, methionine oxidation and the N-terminal protein acetylation were included as dynamic modifications. A maximum of 2 missing cleavages was allowed. The precursor mass tolerance was set to 10 ppm, and the fragment mass tolerance to 0.02 Da. A correction of 1% false discovery rate (FDR) at both peptide and protein levels was applied. Sum of unique + razor peptide intensities were used for protein quantitation, which means that all unique peptides were considered, and shared peptides were used for the protein that had more identified peptides. We considered those proteins as human or mouse proteins in which at least one unique species-specific peptide was identified. On the other hand, those proteins that did not have unique human or mouse peptides, i.e. their origin was unclear, were excluded from further statistical analyses [82].

#### Statistical and biological pathway analyses

Protein intensities were normalized by log2 transformation and the subtraction of median intensities using the software Perseus 1.6.5.0. Principal component analysis of the global proteome was also performed in Perseus 1.6.5.0. To determine statistically significant differences between conditions a two-tailed t-test (*p*-value:0.05) with Benjamini-Hochberg (FDR: 0.05) correction for multiple testing was applied. A cutoff of ± 0.5 was also set for the log2 fold-change (AD/Control) in the post-mortem brain tissue samples. To select proteins significantly altered across the different AD models a one-way ANOVA (*p*-value:0.05) with Benjamini-Hochberg (FDR: 0.05) correction was applied together with the log2 fold-change (AD/Control) cutoff. The Tukey's honest significant difference test was used as a post-hoc test (FDR < 0.05). Additionally, hierarchical clustering analysis was performed using the Pearson correlation distance. For the 1D annotation enrichment analysis of the quantified proteins the Benjamini–Hochberg FDR value of 0.02 was set as a significance limit. The detection of relevant biological pathways was performed in the Metascape [83] incorporating the Gene Ontology Consortium, Reactome Gene Sets, KEGG Pathway, and PANTHER Pathway databases. Enrichment processing included the gene prioritization by evidence counting strategy with a *p*-value cutoff of 0.01.

Finally, data derived from the immunoassays were analyzed using GraphPad Prism 7 software and presented as mean ± S.E.M. Unpaired two-tailed t-test was used to compare the two groups. A *p*-value of < 0.05 was considered significant. For Aβ42 (Fig. 2e), amyloid plaques (H31L21 staining, Fig. 3b), and phospho-tau (AT8 staining, Fig. 3b), data were analyzed via a robust t-test using an M-estimator [84] to account for the presence of a highly influential observation (Cook’s Distance threshold = 0.67 for Aβ42 and = 0.57 for H31L21 and AT8).

### Supplementary Information

**Additional file 1: Figure S1.** Characterization of the iPSC-derived human grafts (HG) in the mouse brain.

**Additional file 2: Table S1.** Global proteome analysis for ADHG.

**Additional file 3: Table S2.** Dysregulated proteins in ADHS, ADHG and ADHPMBT.

**Additional file 4: Table S3.** Global proteome for ADHPMBT.

**Additional file 5: Table S4.** Common and dysregulated proteins across models.

**Additional file 6: Table S5.** Global proteome and dysregulated proteins in ADHG-MT.

**Additional file 7: Table S6.** List of human post-mortem brain samples.

**Additional file 8: Table S7.** Primary and secondary antibodies used for immunocyto- and immunohistochemistry.

**Additional file 9: Table S8.** Primary and secondary antibodies used for Western Blotting.

## Data Availability

The mass spectrometry data of ADHS, ADGH and ADHPMBT have been deposited to the ProteomeXchange Consortium via the PRIDE partner repository with the dataset identifiers PXD012524 and PXD042229. Processed omics data are also available as supplementary material.

## Abbreviations

Aβ: Amyloid-β
AD: Alzheimer’s disease
ADHG: AD hippocampal grafts
ADHG-MT: Mouse tissue adjacent to the AD hippocampal graft
ADHPMBT: AD hippocampal postmortem brain tissue
ADHS: AD hippocampal spheroids
ApoE4: Alipoprotein E
APP: Amyloid precursor protein
BACE1: Beta-secretase 1
CHG: Control hippocampal grafts
Da: Dalton
DAPI: 4′,6-Diamidino-2-phenylindole
FDR: False discovery rate
FGF2: Fibroblast growth factor 2
FTIR: Fourier transform infrared
GFAP: Glial fibrillary acidic protein
GSK3A: Glycogen synthase kinase-3 alpha
GSK3B: Glycogen synthase kinase-3 beta
IBA1: Ionized calcium binding adaptor molecule 1
iPSC: Induced pluripotent stem cells
IT: Injection time
KEGG: Kyoto encyclopedia of genes and genomes
LC–MS/MS: Liquid chromatography-tandem mass spectrometry
MAP2: Microtubule-associated protein 2
MARK1: Microtubule affinity regulating kinase 1
MARK3: Microtubule affinity regulating kinase 3
MSD: Meso scale discovery
NDS: Normal donkey serum
PBS: Phosphate-buffered saline
PBST: Phosphate-buffered saline tween
PD: Proteome discoverer
PET: Positron emission tomography
PFA: Paraformaldehyde
PVDF: Polyvinylidene fluoride
RAG-2: Recombination activation gene 2
RT: Room temperature
TBR1: T-box brain transcription factor 1
TEAB: Triethylammonium bicarbonate
TREM2: Triggering receptors expressed on myeloid cells 2
ZBTB20: Zinc finger and BTB domain containing 20
18F: Flutemetamol tracer
