# Supplementary material for: Proteomic analysis across patient iPSC-based models and human post-mortem hippocampal tissue reveals early cellular dysfunction and progression of Alzheimer’s disease pathogenesis
Source: Acta Neuropathol Commun. 2023 Sep 15;11:150. doi: 10.1186/s40478-023-01649-z (PMC10504768; doi:10.1186/s40478-023-01649-z)

Supplementary figure 1. Characterization of the iPSC-derived human grafts (HG) in the mouse brain

Immunostaining for human Nuclei marker, human cytoplasm marker STEM121, human astrocytic marker Stem123 and marker of proliferation KI67, neuronal progenitor marker DCX, astrocyte marker GFAP and microglial marker IBA1 in control and AD iPSC-derived human grafts.

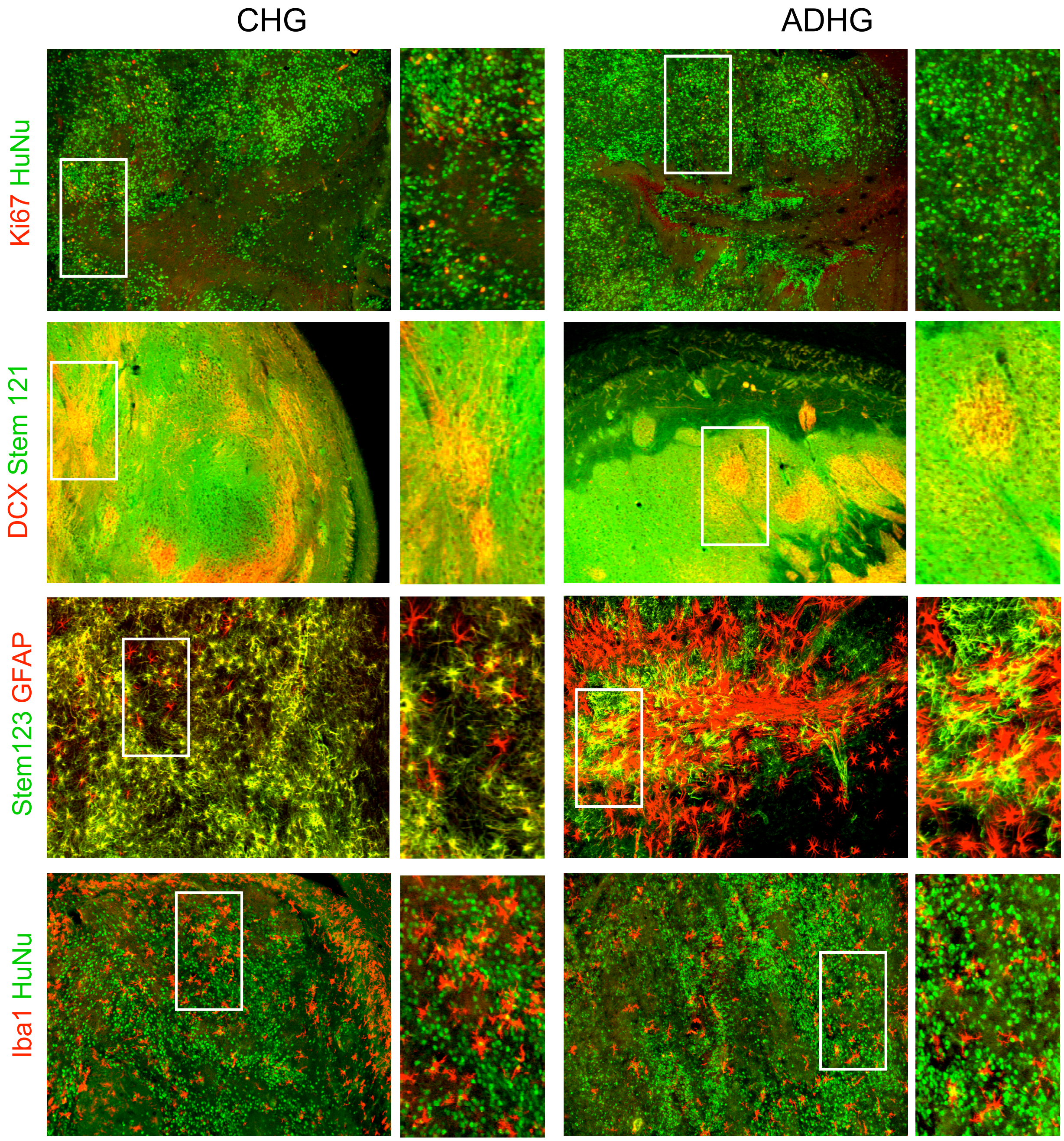

Supplement: Supplementary file 1 — Additional file 1: Figure S1. Characterization of the iPSC-derived human grafts (HG) in the mouse brain. [file 40478_2023_1649_MOESM1_ESM.pdf]
